# The pathophysiological role of mitochondrial oxidative stress in lung diseases

**DOI:** 10.1186/s12967-017-1306-5

**Published:** 2017-10-13

**Authors:** Xiaojing Liu, Zhihong Chen

**Affiliations:** 1 Respiratory Division of Zhongshan Hospital, Shanghai Institute of Respiratory Diseases, Fudan University, No. 180 Fenglin Road, Shanghai, 200032 China; 2 Geriatric Department, Shanghai Jiao Tong University Affiliated Sixth People’s Hospital, No 600 Yishan Road, Shanghai, China

**Keywords:** Mitochondrial DNA, Reactive oxygen species, Antioxidant agents

## Abstract

Mitochondria are critically involved in reactive oxygen species (ROS)-dependent lung diseases, such as lung fibrosis, asbestos, chronic airway diseases and lung cancer. Mitochondrial DNA (mtDNA) encodes mitochondrial proteins and is more sensitive to oxidants than nuclear DNA. Damage to mtDNA causes mitochondrial dysfunction, including electron transport chain impairment and mitochondrial membrane potential loss. Furthermore, damaged mtDNA also acts as a damage-associated molecular pattern (DAMP) that drives inflammatory and immune responses. In this review, crosstalk among alveolar epithelial cells, alveolar macrophages and mitochondria is examined. ROS-related transcription factors and downstream cell signaling pathways are also discussed. We conclude that targeting oxidative stress with antioxidant agents, such as thiol molecules, polyphenols and superoxide dismutase (SOD), and promoting mitochondrial biogenesis should be considered as novel strategies for treating lung diseases that currently have no effective treatment options.

## Background

The lungs exist in a high-oxygen environment. Their large surface area and high blood supply make the lungs susceptible to injuries mediated by oxidative stress [1–3]. Reactive oxygen species (ROS) are generated both endogenously and exogenously. During exogenous ROS exposure, environmental gases, such as aldehydes/carbonyls, NO_2_, SO_2_, CO, and airborne particulate matters, as well as cigarette smoke, can cause oxidative stress and trigger inflammatory responses in the lungs [4, 5]. In addition, impaired antioxidant defense systems in lung epithelial cells, macrophages and other inflammatory cells can lead to high levels of endogenous ROS in tissues.

Mitochondria are centrally involved in ROS-dependent pathways. Mitochondrial dysfunction plays a crucial role in bioenergetics metabolism and non-energetics pathogenesis in many lung diseases [6, 7]. The mitochondrial genome acts as a sentinel factor governing the cytotoxic response of lung cells to oxidant stress [8, 9]. In this review, we highlight how ROS-induced mitochondrial dysfunction and damaged mitochondrial DNA (mtDNA) fragments are involved in the pathobiology of various degenerative lung diseases, pulmonary fibrosis, and lung tumorigenesis. These findings imply that antioxidants can detoxify free radicals and modulate oxygen redox reactions to enhance glutathione (GSH) biosynthesis, improve chromatin remodeling and decrease lung inflammation. Various dietary and pharmacological approaches for increasing lung antioxidant levels and their beneficial effects on a variety of lung diseases will be discussed.

## Exogenous and endogenous ROS

Reactive oxygen species, such as superoxide anions (O_2_^·−^) and hydroxyl radicals (^·^OH), are unstable molecules that can initiate oxidation via unpaired electrons. The O_2_^·−^ radical can either react with NO to form a highly reactive peroxynitrite molecule (ONOO^−^) or be rapidly converted to hydrogen peroxidase (H_2_O_2_) by superoxide dismutase (SOD). H_2_O_2_ can be converted into the damaging ^·^OH in the presence of Fe^2+^; this process is called the Fenton reaction.^·^OH can also be generated from O_2_^·−^ via the Haber–Weiss reaction. In the presence of chloride (Cl^−^) and bromide (Br^−^) ions, H_2_O_2_ is catalyzed by heme peroxidases or myeloperoxidase to form hypochlorous acid (HOCl) and hypobromous acid (HOBr), which are very damaging oxidants [10]. High levels of ROS have been implicated in the oxidation of proteins, lipids and DNA; this oxidation can result in tissue injury and inflammatory responses [11].

As previously mentioned, the key sources of ROS are exogenous exposure and endogenous release from inflammatory cells, macrophages and epithelial and endothelial cells. In addition, the mitochondria in these cells are central to ROS production. Previous data revealed that alveolar macrophages (AMs) exposed to asbestos fibers can produce H_2_O_2_. ROS production can be reduced by knocking down the iron-sulfur protein of complex III in the mitochondrial electron transport chain (ETC), a major site of ROS production. This study implies that the mitochondrial ETC plays a critical role in ROS production [12, 13]. In the basal status, cytosol in pulmonary artery smooth muscle cells (PASMCs) has a low oxidation state, but the mitochondrial matrix has a high oxidation state [14]. When subjected to acute hypoxia, the oxidation status in the cytosol increases, whereas the oxidation status in the mitochondrial matrix decreases. These actions imply that ROS exit the cytosol to initiate redox signaling. ROS are also generated when the antioxidant defense system is downregulated. This system includes catalase, GSH and SOD. Intratracheal catalase administration to asbestos-treated mice prevented pulmonary fibrosis by inhibiting H_2_O_2_ production [15]. In another study, GSH was reduced in the epithelial lining fluid and fibrotic foci in idiopathic pulmonary fibrosis (IPF) lungs [16]. In addition, SOD knockout mice had more fibrosis after exposure to environmental toxins [17]. Therefore, imbalance of the antioxidant and oxidant system plays a role in the pathogenesis of ROS-induced lung diseases.

## Mitochondrial metabolism and functions

Mitochondria are double membrane-bound organelles that exist in all eukaryotic organisms. The essential cellular function of mitochondria is to generate energy in the form of adenosine triphosphate (ATP). This function makes mitochondria the “powerhouse” of the cell. Mitochondria are commonly between 0.75 and 3 μm in diameter but vary considerably in size and structure. They can be viewed by using only an electron microscope. The number of mitochondria in a cell varies widely from one to several thousand, and this number depends on the tissue type and organism [18, 19].

Mitochondria are composed of several compartments, including the outer membrane, the intermembrane space, the inner membrane, the cristae and the matrix. Each part has different functions. The production of ATP relies on many proteins, including nicotinamide adenine dinucleotide (NADH) dehydrogenase, cytochrome c reductase, and cytochrome c oxidase, located in the inner membrane. These proteins oxidize pyruvate, glucose, and NADH in the cytosol and perform the energy transfer. This type of cellular respiration relies on oxygen and is known as aerobic respiration. In addition to supplying ATP, mitochondria are also involved in other tasks, such as signaling, cellular differentiation, cell cycle regulation and cell growth. Although the process is very efficient, some high-energy electrons leak from the respiratory chain and can form ROS [20]. This process was thought to result in significant oxidative stress in mitochondria with high mtDNA mutation rates, and this oxidative stress could promote the pathology of degenerative diseases and tumorigenesis [21].

## ROS-induced mtDNA damage

The human mitochondrial genome is a circular DNA molecule of approximately 16 kilobases. It encodes 37 genes: 13 genes for subunits of respiratory complexes I, III, IV and V; 22 genes for mitochondrial tRNA; and 2 genes for rRNA. One mitochondrion can contain two to ten copies of its DNA. mtDNA is present in multiple copies (~ 100) per cell and encodes approximately 3% of all mitochondrial proteins [22]. These proteins are essential for regulating mtDNA-associated proteins, including 8-oxoguanine glycosylase (OGG1), mitochondrial aconitase (ACO2), mitochondrial transcription factor A (Tfam), and others [23]. mtDNA is approximately 50-fold more sensitive to oxidative damage than nuclear DNA due to its proximity to the ETC, lack of a histone protective shield covering the mtDNA and limited DNA repair mechanisms [24–26].

**Fig. 1:**
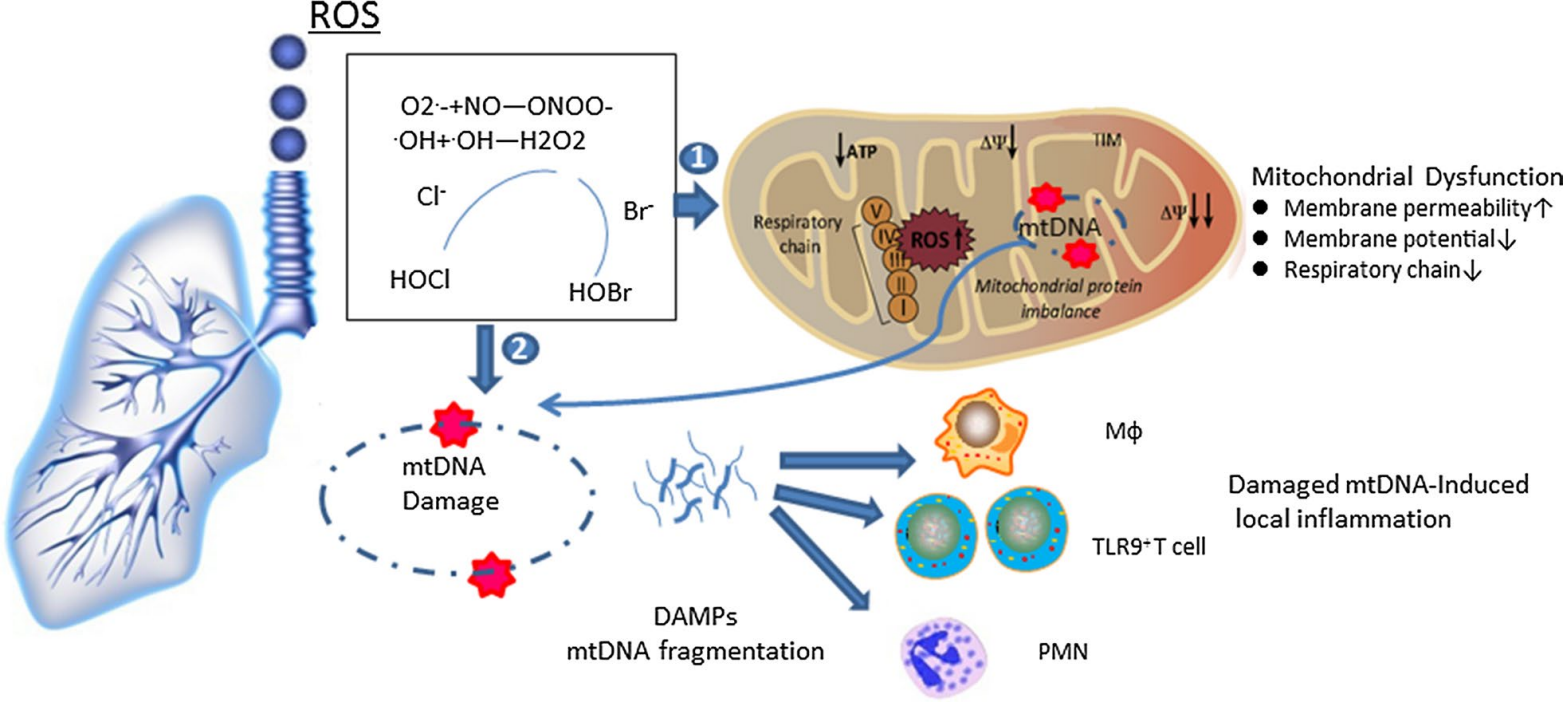
Proposed model for mitochondria-centered pathogenesis in ROS-induced lung diseases. Reactive oxygen species (ROS), commonly from exogenous exposure to smoke and air pollution, can be inhaled by the lungs. The superoxide anion radical (O_2_^·−^) can react with NO to form the highly reactive peroxynitrite molecule (ONOO^−^). In the presence of chloride (Cl^−^) and bromide (Br^−^) ions, the very damaging oxidants hypochlorous acid (HOCl) and hypobromous acid (HOBr) are generated from hydrogen peroxidase (H_2_O_2_). (1) ROS-induced mitochondrial dysfunction: when the concentration of ROS increases in mitochondria, the electron chain transfer (ETC) complex becomes defective and leads to mitochondrial membrane potential loss and membrane permeability increases. (2) Mitochondrial DNA (mtDNA)-associated immune response: damaged mtDNA fragments are released from mitochondria. The immunogenic particles can recruit various immune cells, such as macrophages, TLR9^+^-T cells and neutrophils, to the damaged area to initiate inflammatory and immunological reactions

mtDNA variants within cells can affect both energy and non-energy pathways (complement, inflammatory, and apoptotic); these effects have shifted the paradigm regarding the role of mitochondria to one beyond merely energy production [27–29]. For example, mtDNA damage causes the loss of mitochondrial membrane potential (ΔΨm) and then influences ETC efficiency; these changes contribute to increased permeability in the outer mitochondrial membrane [30, 31]. In addition, pro-apoptogenic agents are commonly released after mtDNA damage and drive disease formation, aging, and tumorigenesis. For example, Bax and Bak can induce outer mitochondrial membrane permeabilization and then cause the release of apoptogenic molecules from the mitochondria to promote caspase activation [32–34]. Oxidation-induced mtDNA damage can both trigger the death of the affected cell and transmit signals to “alarm” neighboring and itinerant cells (Fig. 1).

## Damaged mtDNA acts as a damage-associated molecular pattern (DAMP)

Instead of following nuclear genetic principles, mtDNA is maternally inherited. In addition, mutations in the key genes involved in mitochondrial energy-generating oxidative phosphorylation account for the complex clinical-pathological features of many common degenerative and metabolic diseases. After injury, mtDNA fragments accumulate in autolysosomes where DNase II is capable of degrading these fragments into biological insignificance. However, some studies suggest that mtDNA fragments play a pathophysiological role in lung disease. For instance, oxidatively damaged mtDNA fragments can upregulate mitophagy after they are exported from the organelle. mtDNA also acts as a DAMP to initiate immunologic responses [35]. In severely injured or critically ill human patients, mtDNA accumulates in the circulation. It functions as a DAMP and causes both TLR9-dependent activation of the innate immune system and NALP3-dependent activation of caspase 1. The activation of both TLR9 and NALP3 can initiate downstream pro-inflammatory signaling [36, 37].

Mitochondrial transcription factor A (TFAM) is an essential mitochondria transcriptor located in the nucleus. The TFAM-mtDNA complex augments mtDNA immunogenicity, which acts as a DAMP to activate plasmacytoid dendritic cell (pDC)-like macrophages and recruit them from the lymph nodes [32, 38]. These pDC-like macrophages express CCR2 and are recruited to the lungs by CC chemokine ligand-2 (CCL2). They also facilitate the clearance of damaged cells in acute lung injury (ALI). Taken together, these findings indicate that damaged mtDNA is involved in the pathogenesis of many lung diseases [39] (Fig. 1).

## Alveolar epithelial cell (AEC) and AM dysfunction induced by mtDNA damage

Alveolar epithelial cell apoptosis is a key pathophysiological event that hinders normal lung repair and thus promotes pulmonary fibrosis [40]. Patients with IPF and animal models of asbestos- and silica-induced pulmonary fibrosis have severe lung epithelial cell injury and apoptosis that are induced in part by mitochondrial ROS [41]. Recently, two groups have established that PTEN-induced putative kinase 1 (PINK1) deficiency impairs AEC mitochondrial function in patients with IPF [42, 43]. Moreover, the pro-fibrotic cytokine TGF-β may protect lung epithelial cells by promoting PINK1 expression and attenuating AEC apoptosis, which drives lung fibrosis. The evidence implies that oxidative stress induces mtDNA damage and thus promotes AEC apoptosis and pulmonary fibrosis [44].

Although epithelial cells are very important in lung fibrosis and degenerative diseases, other cell types, such as vascular endothelial cells and macrophages, are also likely important. Carter et al. suggested that mitochondrial ROS from AMs play a key role in mediating asbestosis. In this study, mitochondrial Rac-1 levels were elevated in AMs from patients with asbestosis. In addition, Rac-1 augmented asbestos-induced H_2_O_2_ production in AMs. These data indicate that H_2_O_2_ production via electron transfer may activate cellular injury pathways that promote asbestosis [45].

## Transcription factor and cell signaling pathway changes after mtDNA damage

### Ogg1

OGG1 is a DNA glycosylase enzyme. It can be found in bacterial, archaeal and eukaryotic species and is involved in base excision repair. OGG1 is the primary enzyme responsible for the excision of 8-oxoguanine (8-oxoG), a mutagenic base byproduct that results from ROS exposure. OGG1 is a bifunctional glycosylase because it can both cleave the glycosidic bond of the mutagenic lesion and cause a strand break in the DNA backbone [23]. The activity of OGG1 is over three-fold higher in mitochondria than in the nucleus. Mitochondria-targeted OGG1 (mt-OGG1) overexpression prevents mitochondria-regulated apoptosis caused by oxidative stress and AEC apoptosis caused by asbestos exposure. mt-OGG1 overexpression promotes AEC survival despite high levels of asbestos-induced mitochondrial ROS stress [46].

### Aco2

Aconitase (ACO) belongs to the aconitase isomerase family. It is an enzyme that catalyzes the conversion of citrate to isocitrate via cis-aconitate in the second step of the tricarboxylic acid (TCA) cycle. ACO is encoded in the nucleus and functions in the mitochondria. The mitochondrial form of aconitase, ACO2, is correlated with many diseases because it is directly involved the central metabolic pathway, which includes converting glucose into ATP. Decreased ACO2 expression levels in gastric cancer cells have been associated with a poor prognosis. ACO2 inactivation has also been associated with decreased lifespan in yeast and progressive neurodegenerative diseases in humans; these data support the protective role of ACO2. The translational significance of ACO2 in preserving mtDNA integrity and preventing pulmonary fibrosis, however, still needs to be assessed [47, 48].

### NOX-Nrf2

NOX is a nicotinamide adenine dinucleotide phosphate (NADPH) oxidase. It can be found in the plasma membrane and in the membranes of phagosomes, which can engulf microorganisms, in neutrophils. NOX generates superoxide by transferring electrons from NADPH inside the cell across the membrane and coupling these to molecular oxygen to produce O_2_^·−^, which is a reactive free radical. Superoxide kills bacteria and fungi by mechanisms that are not yet fully understood. However, superoxide may inactivate critical metabolic enzymes, initiate lipid peroxidation and liberate redox-active iron; these actions may generate indiscriminate oxidants, such as ^·^OH [49]. Studies of human IPF lung tissues have demonstrated that NOX4 is related to senescence and apoptosis resistance. Genetic and pharmacological NOX4 inhibition attenuated the senescent and apoptosis-resistant myofibroblast phenotype in a model of established fibrosis in older mice and led to the reversal of persistent fibrosis.

Nuclear factor erythroid 2-related factor (2Nrf2) is a transcription factor that is encoded by the NFE2L2 gene. Nrf2 regulates the expression of antioxidant proteins that protect against oxidative damage caused by injury and inflammation [50]. Under oxidative stress, Nrf2 travels to the nucleus where it binds to the antioxidant response elements (AREs) of gene promoters; then, instead of degradation, Nrf2 initiates the transcription of antioxidative genes and their proteins [51]. Several recent studies have highlighted the importance of Nrf2 in regulating pulmonary fibrosis. Nrf2 knockout mice are more sensitive to bleomycin- and paraquat-induced pulmonary fibrosis than wild-type animals [52]. In a study of primary lung fibroblasts cultured from healthy or IPF patients, decreased Nrf2 expression levels were associated with a myofibroblast phenotype, whereas Nrf2 activation increased antioxidant defense and myofibroblastic dedifferentiation in IPF fibroblasts [53]. Imbalances between NOX and Nrf2 result in increased oxidation and impaired antioxidation capacity. Thus, correcting NOX/Nrf2 redox imbalance may be an important therapeutic target for treating ROS-related diseases [54–56].

### p53

p53 has been described as “the guardian of the genome” because it preserves stability by preventing genome mutation. p53 has many anticancer functions and plays a role in apoptosis, genomic stability, and angiogenesis inhibition [57, 58]. p53 likely has relations with the genes OGG1 and ACO2. It is also involved in the pathobiology of degenerative diseases. For example, p53 can reduce ACO2 gene expression levels. Camptothecin (CPT) treatment induced p53 expression while reducing mACON protein biosynthesis, which could be blocked by cyclic pifithrin-a, an inhibitor of p53 transcriptional activity. The process may have little relation with the putative consensus p53 response elements found within the mACON promoter [59]. In asbestos-induced lung fibrosis in mice, p53 activation is required for oxidant-induced apoptosis in OGG1-deficient human fibroblasts. p53 sensitizes HepG2 cells to oxidative stress by diminishing the potential of the electron transport chain to increase ATP production, which leads to the mtDNA depletion [60]. Taken together, these data support a key role for p53 in modulating AEC mtDNA damage in pro-fibrotic lung responses following asbestos exposure.

### Pai-1/nlrp3

Plasminogen activator inhibitor-1 (PAI-1), also known as endothelial plasminogen activator inhibitor, is a protein encoded by the SERPINE1 gene in humans. Increased PAI-1 levels are a risk factor for thrombosis and atherosclerosis. PAI-1 is a serine protease inhibitor (serpin) that functions as the principal inhibitor of tissue plasminogen activator (tPA) and urokinase (uPA), which activate plasminogen to induce fibrinolysis. In addition, PAI-1 inhibits the activity of matrix metalloproteinases, which play a crucial role in malignant cell invasion through the basal lamina [61]. Airway epithelial type 2 (AT2) cells from patients with IPF and chronic obstructive pulmonary disease (COPD) had reduced expression levels of E-cadherin and zona occludens-1, whereas collagen-I and α-smooth muscle actin expression levels were increased in parallel with increased PAI-1 and reduced uPA expression levels [62, 63]. These studies suggest that the induction of PAI-1 and the inhibition of uPA during fibrotic lung injury may promote epithelial-mesenchymal transition (EMT) in AT2 cells. PAI-1 knockdown and overexpression studies in cultured fibroblast have confirmed the inverse relationship between PAI-1 activation and collagen production. PAI-1 depletion in fibroblasts yields a cell that produces activated collagen and is resistant to senescence/apoptosis; however, activated PAI-1 upregulates AT2 cell apoptosis, which is crucial for the propagation of lung fibrosis. But there are still needs for further evidence to support the contention that apoptosis and EMT, as a result of increased PAI-1 by AT2 cells, contributes to fibrogenesis.

NLRP3 is a component of the inflammasome and is mostly expressed in macrophages. Its function is to detect damaged cell products, such as extracellular ATP and crystalline uric acid. Once NLRP3 is activated, it triggers an immune response. Mutations in the NLRP3 gene are associated with a number of organ-specific autoimmune diseases. As part of the innate immune system, NLRP3 serves as a pathogen-associated molecular pattern (PAMP) [64]. The NLRP3 inflammasome appears to be activated by changes in intracellular potassium levels; these changes are usually caused by potassium efflux from mechanosensitive ion channels located in the cell membrane [65]. However, how ion channel opening is linked to NLRP3 activation remains elusive. Vimentin promotes ALI, alveolar epithelial barrier permeability and lung fibrosis by regulating NLRP3 inflammasome signaling. Without NLRP3 inflammasome signaling, bone marrow chimeric mice lacking vimentin have decreased lung fibrosis when induced by asbestosis [66]. It is still not known how it is regulated during NLRP3-mediated inflammasome formation. NLRP3 is likely correlated with ROS-mediated immune responses and inflammation [67], but the precise mechanisms of such regulation have not been determined.

## ROS-related lung diseases

### ROS and lung fibrosis

Mitochondrial ROS generation has been associated with increased cellular oxidative stress and apoptosis in AECs. In addition, lung specimens from IPF patients show increased expression levels of iNOS [68, 69]. Oxidized lipids and proteins have been identified in the exhaled air, bronchoalveolar lavage (BAL) fluid, and lung tissues of patients with fibrotic lung disease [70]. Oxidized lipids and proteins have also been identified in a bleomycin-induced pulmonary fibrosis mouse model [71]. These findings suggest that patients with IPF have increases in both oxidative and nitrosative stress. Oxidants may play a role in pulmonary fibrosis by affecting the production of cytokines and growth factors, such as TGF-β, which is a key regulator of the aberrant repair mechanisms that are characteristic of many fibrotic diseases, including IPF. N-acetyl-l-cysteine (NAC), which is an antioxidant substance, attenuates bleomycin-induced fibrosis in rodents and increases lung levels of GSH.

### ROS and asbestos

Asbestos fibers can induce mitochondrial ROS production in lung epithelial cells and macrophages [40]. After exposure, asbestos fibers are internalized by AECs. This process can lead to the production of iron-derived ROS, mtDNA damage and intrinsic apoptosis; evidence of these effects includes decreased ΔΨm, mitochondrial cytochrome c release into the cytosol, and caspase-9 and caspase-3 activation. Mossman et al. showed that activated protein kinase delta (PKCδ) migrated to the mitochondria of lung epithelial cells both in vitro and in vivo upon exposure to asbestos. This action depended on pro-apoptotic Bim activation. In addition, PKCδ activation is crucial for promoting asbestos-induced mitochondria-regulated apoptosis and fibrosis [72]. Antioxidants and iron chelators can attenuate fibrosis induced by bleomycin or asbestos in rodent models. Taken together, mitochondrial ROS production and PKCδ activation following asbestos exposure appear to be important for intrinsic lung epithelial cell apoptosis [73, 74].

### ROS and airway diseases

Four principal mechanisms are responsible for the alterations observed in COPD: oxidative stress, inflammation, protease-antiprotease imbalance and apoptosis [75]. The intricacies of each mechanism contribute to various disease presentations. Among these mechanisms, oxidative stress plays a critical role in the pathogenesis of COPD because of its ability to trigger and exacerbate the three other mechanisms [76, 77]. Oxidants also promote inflammation by activating NF-κB, which regulates the expression of multiple inflammatory genes, including IL-8 and TNF-α, that are important in COPD. Markers of oxidative stress (e.g., H_2_O_2_ and NO) have been found in the epithelial lining fluid, breath and urine of patients with COPD [78]. Circulating neutrophils from patients with COPD produced increased levels of O_2_^·−^ and adhesion molecules. Lipid peroxidation products, such as thiobarbituric acid-reactive substances (TBARs), conjugated dienes of linoleic acid and F2-isoprostane, are significantly increased in the plasma of patients with acute exacerbations of chronic bronchitis [79]. Imbalance between proteases and endogenous antiproteases also plays an important role in the pathogenesis of COPD. Oxidants can potentiate the effects of proteases on COPD through activating these enzymes. Oxidative stress and chronic inflammation interact and act as synergistic factors to promote systemic impairments (e.g., weight loss and skeletal muscle dysfunction) in patients with COPD.

Asthma is a chronic inflammatory airway disease, and oxidative stress may be involved in its pathogenesis. Chronic airway inflammation, airway smooth muscle contraction and bronchial hyperreactivity are three major characteristics of the pathogenesis of asthma [80–82]. Considerable evidence has shown that ROS are involved in these three processes. For example, eosinophil peroxidase (EPO) and myeloperoxidase (MPO) levels are increased in the peripheral blood, induced sputum, and BAL fluid from patients with stable asthma. Recently, high levels of many direct and indirect oxidative stress markers, including malondialdehyde, TBARs, and H_2_O_2_, have been found in the urine, plasma, sputum, BAL fluid, and lung tissues of patients with asthma [83, 84]. ROS are widely involved in airway smooth muscle contraction, adrenergic receptor function impairment, decreased epithelial cilia numbers and function, increased mucus production and increased vascular permeability. In addition, the overproduction of ROS or suppression of the protective system also results in bronchial hyperreactivity, which is characteristic of asthma. Animal models have shown that ROS contribute to airway hyperresponsiveness by decreasing mucociliary clearance and by increasing the vagal tone via oxidant-sensitive β-adrenergic receptor damage [85–87].

### ROS and ALI/acute respiratory distress syndrome (ARDS)

In patients with ALI/ARDS, there are many potential sources of ROS, including inflammatory cells (neutrophils, monocytes, and macrophages) and parenchymal cells (endothelial and epithelial cells, fibroblasts, and myocytes). Markers of ROS and reactive nitrogen species (RNS) formation are increased significantly in the lung lining fluid from patients with ARDS [88]. H_2_O_2_ has also been directly detected in the exhaled breath of patients with ARDS. High levels of H_2_O_2_ are found in exhaled air and urine of patients with ARDS; these patients also have high circulating levels of 4-hydroxy-2-nonenal (4-HNE). In addition, the effectiveness of the antioxidant defense system has been shown to decrease with increasing levels of ROS [89].

Plasma inflammasomes, such as cytokines and PAMPs, are involved in the pathogenesis of patients with critical illnesses, such as ALI/ARDS [90]. Recent studies suggest that mtDNA is released from mitochondria into the cytosol as a mitochondrial DAMP and serves as a potent inflammasome activator. In addition, circulating levels of mtDNA are associated with disease severity or mortality in ICU patients [91]. Circulating cell-free mtDNA levels were higher in ICU patients who died within 28 days of medical ICU admission, as well as in ICU patients with sepsis or ARDS. Circulating cell-free mtDNA levels were also higher in patients with severe trauma or sepsis in the emergency room [92]. These results imply that circulating mtDNA could serve as a viable plasma biomarker to represent the exaggerated systemic inflammatory response levels observed in patients with sepsis or ARDS.

### ROS and lung cancer

**Fig. 2:**
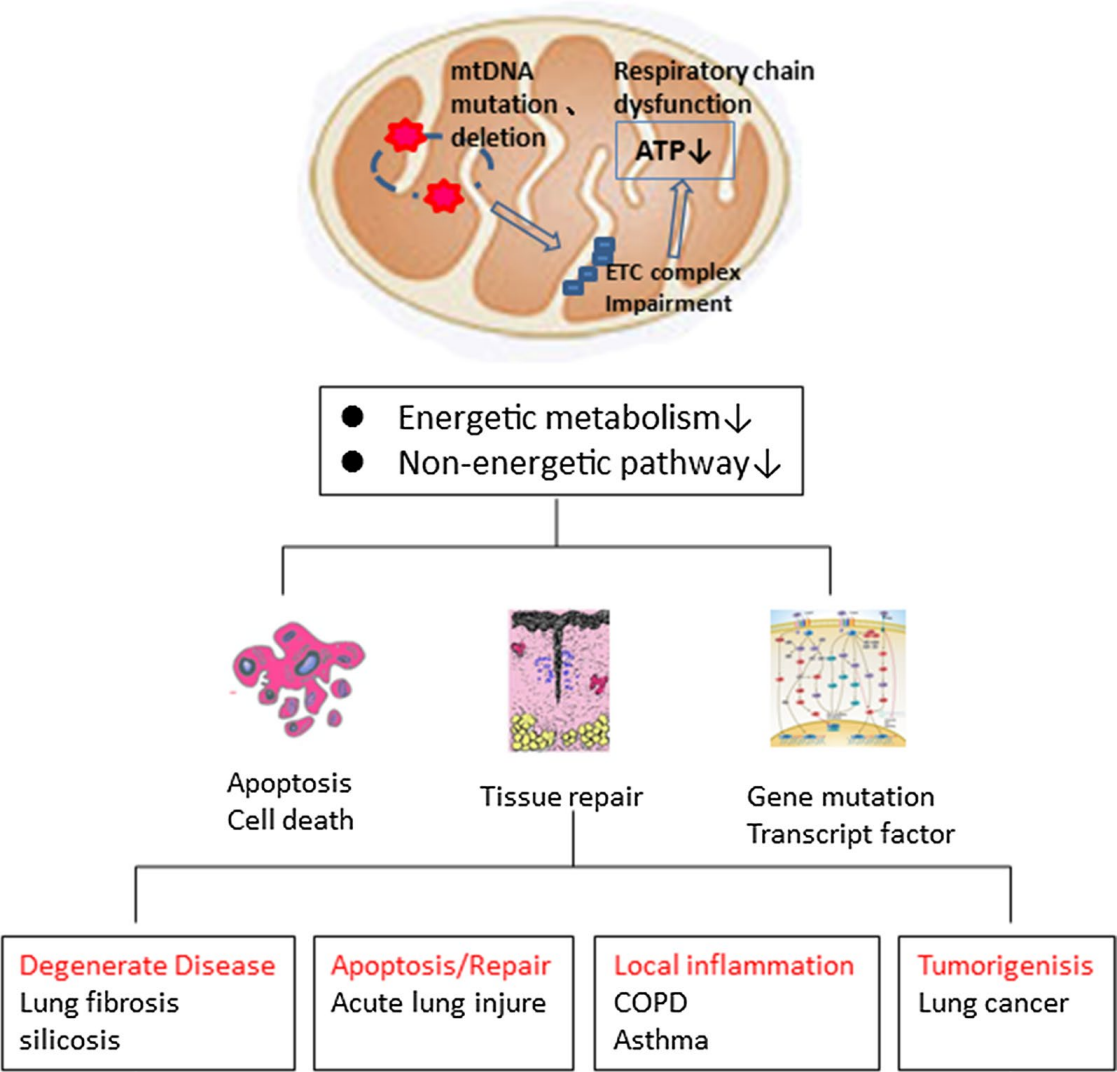
Mitochondrial dysfunction and diverse lung diseases. Mitochondrial dysfunction and mitochondrial DNA mutations and deletions can cause defects in energy metabolism and impair non-energetic pathways. Mitochondrial dysfunction is also associated with lung alveolar epithelial cell and alveolar macrophage apoptosis, which hinders lung repair and influences the expression of various reactive oxygen species-related genes. Thus, mitochondrial dysfunction can induce diverse degenerative lung diseases

Tumor cells require an ample amount of ATP to synthesize bioactive compounds, such as lipids, proteins and nucleotides, for rapid cell proliferation. Mitochondria play a role in cell proliferation, and it has been shown that interference with the oxidative process can cause cell cycle arrest. Oxidative stress is involved in the malignant transformation process, which includes initiation, promotion and progression. During the initiation stage, ROS may cause DNA damage via gene mutations and structural alterations to the DNA. In the promotion stage, ROS can promote abnormal gene expression, block cell-to-cell communication, and modify second-messenger systems. These actions result in an increase in cell proliferation or a decrease in apoptosis in the affected cell population. Finally, oxidative stress may participate in the progression stage of cancer by further altering DNA in the affected cell population. A research study examining 41 human lung, bladder, and head and neck tumors revealed that mutations were 19–220 times more frequent in mtDNA than in nDNA. According to subgroup analyses, over 40% of patients with lung cancer have mutations in their mtDNA; this finding emphasizes the importance of mtDNA mutations in lung cancer [93]. MtDNA mutations can compromise ETC function and alter metabolism. These actions can accelerate aerobic glycolysis in metastatic progression. Furthermore, severe mtDNA damage promotes mitochondrial genome deletion [94]. These studies demonstrate that mtDNA damage and mutations occur in lung cancers. Preserving mtDNA integrity may be an innovative preventative therapeutic target [95] (Fig. 2).

## Therapeutic intervention with antioxidants

Many studies have shown that oxidative stress is involved in the pathogenesis of various lung diseases, such as IPF, asbestosis, COPD, asthma and lung cancer. Thus, antioxidant intervention seems to be an ideal method for relieving symptoms and preventing radical progression [96–99]. Increasing the endogenous antioxidant enzyme defenses and enhancing the non-enzymatic defenses through dietary and pharmacological means will be discussed (Table 1).

**Table 1:** Therapeutic antioxidant use in lung diseases

Classification	Antioxidant compounds	Potential lung disease applications
Dietary vitamins	Ascorbic acid Vitamin E β-carotene Selenium All-trans retinoic acid (ATRA)	Asthma, COPD, cigarette smoking, emphysema
Thiols	Glutathione (GSH)	COPD, asthma
	N-acetyl-l-cysteine (NAC) N-acystelyn (NAL) N-iso-butyryl-cysteine (NIC)	IPF, silicosis, ALI/ARDS, chronic bronchitis
	Erdosteine Fudosteine	COPD, chronic bronchitis, cigarette smoking
	Thioredoxin (MOL-294)	Lung cancer
Enzyme mimetics	SOD	ALI/ARDS, COPD, cigarette smoking, asthma
	Ebselen	COPD, asthma
PDE4 inhibitors	Roflumilast Cilomilast	COPD, asthma, chronic bronchitis
Polyphenols	Resveratrol Curcumin Quercetin Lycopene Acai	COPD ALI/ARDS

### Dietary antioxidant vitamins

Low levels of ascorbic acid, vitamin E, β-carotene and selenium have been observed in the serum of COPD patients [100, 101]. Moreover, decreased vitamin E and vitamin C levels were reported in leukocytes and BAL fluid from smokers [102, 103]. Cigarette smoke-induced plasma lipid peroxidation in vitro is decreased by ascorbate. Although these vitamins have been investigated in many diseases, the results are not consistent [104–106]. Population studies suggest that the intake of antioxidant vitamins can improve lung function. Dietary antioxidant intake can prevent the development of obstructive airway disease. On the other hand, studies also show no clinical benefit of supplemental vitamin C or E intake compared to current standard therapies for mild to moderate asthma [107]. Robust clinical trials using dietary antioxidant vitamins are urgently needed to address the beneficial effects of these antioxidants in lung diseases.

### Gsh

GSH is present at high concentrations in the epithelial lining fluid (ELF). In addition, GSH protects against oxidants in the extracellular milieu [108]. As such, a direct increase in lung cellular levels of GSH would be a logical approach for treating ROS-induced lung diseases. In fact, extracellular GSH augmentation has been attempted through the intravenous administration of GSH, oral ingestion of GSH, and aerosol inhalation of nebulized GSH. However, these routes of administration have undesirable effects. Therefore, direct GSH therapy may not be an appropriate way to increase GSH levels in lung ELF. This problem with direct GSH therapy may be due to the bioavailability of GSH or the pH and osmolality of particular micro-environments. Thus, neutralizing the pH, providing GSH in salt form, maintaining isotonicity or altering the administration route (e.g., liposome-encapsulated delivery) may be useful [109, 110].

### Nac

N-acetylcysteine (NAC) is a precursor of GSH. NAC is de-acetylated to cysteine in the gut following oral administration. This process is important for increasing intracellular GSH levels in vivo in the lungs. Recent studies using NAC have shown its protective benefits against oxidative stress both in vitro and in vivo. COPD patients given 600 mg of NAC orally once daily had a lower risk of exacerbations and better symptom scores than COPD patients given a placebo [111]. NAC is also used as a mucolytic agent to reduce mucus viscosity and improve mucociliary clearance. A Cochrane systematic review was used to evaluate the effects of mucolytic agent treatment in COPD patients. Exacerbations were 79% lower per patient per year for patients receiving mucolytic treatment than for patients receiving a placebo. Even though a significant reduction in exacerbation may occur due to altered mucus production and antibacterial or immunostimulatory effects, the exact mechanism is still unknown [112].

In contrast, the recently published BRONCUS study evaluating NAC treatment (600 mg p.o., daily) over a 3-year period in 523 COPD patients showed that NAC (600 mg p.o., daily) was ineffective at both halting lung function decline and preventing exacerbations in COPD [113]. The variability in current studies reflects the fact that the NAC dose is not high enough. NAC is hydrolyzed in biological systems after administration, and the measured plasma concentrations of the drug are low. Thus, a new drug with greater bioavailability than NAC could be synthesized and used as a more effective treatment for chronic bronchitis.

### Erdosteine

Erdosteine is a new thiol compound that, in addition to its mucoactive properties, acts as an antioxidant. Erdosteine contains two blocked sulfhydryl groups that are released following first-pass metabolism. The three active metabolites exhibit mucolytic and free radical scavenging activities. Erdosteine improves expectoration by modulating mucus production and viscosity and increasing mucociliary transport. It also inhibits the effects of free radicals produced by cigarette smoke.

In the “Equalife” randomized placebo-controlled clinical study, erdosteine was given orally 300 mg b.i.d. for 8 months [114]. Patients receiving erdosteine had fewer exacerbations and spent fewer days in the hospital. Moreover, these patients showed no reduction in lung function during the trial, but their health-related quality of life was significantly improved. Similar to the Cochrane review meta-analysis on mucolytic reagents in chronic bronchitis, it is not clear whether the quality of life benefits were due to antioxidant effects or mucolytic effects.

### Sod

Superoxide can cause many types of cell damage if not regulated. SOD is an enzyme that alternately catalyzes the dismutation of the O_2_^·−^ radical into either ordinary molecular oxygen or H_2_O_2_. Thus, SOD is important in antioxidant defense in nearly all living cells [115]. SODs are a group of metalloproteins with unknown function and include the Cu/Zn type, which binds both copper and zinc; the Fe and Mn types and the Ni type. Low SOD3 activity may be involved in some lung diseases, such as ARDS or COPD [116]. A number of SOD mimetics based on organomanganese complexes have been developed. These mimetics retain their antioxidant properties in vivo. For example, the metalloporphyrin-based compounds AEOL10113 and AEOL10150 have been studied in models of airway inflammation. AEOL10113 inhibited both airway inflammation and bronchial hyperreactivity in an ovalbumin-challenged model of airway inflammation. In another study, AEOL10150 inhibited lung inflammation induced by cigarette smoke [117, 118]. These results imply that SOD can improve ROS-induced lung diseases.

### Phosphodiesterase type 4 (PDE4)

PDE4 inhibitors are drugs used to block the PDE4 degradation of cyclic adenosine monophosphate (cAMP). The PDE4 family of enzymes comprise the most prevalent PDEs in immune cells, which is where they are primarily responsible for hydrolyzing cAMP.

High levels of cAMP in neutrophils block the assembly of NADPH oxidase to inhibit superoxide production. PDE4 inhibitors have a broad anti-inflammatory profile because they inhibit the expression of a variety of cytokines, such as TNF and MIP-1 [119, 120]. The clinical benefits of PDE4 inhibitors have been demonstrated in COPD [121]. In 2010, the PDE4 inhibitor roflumilast (trade names: Daxas or Daliresp) was approved in the EU for treating severe COPD associated with chronic bronchitis. In March 2011, Daliresp gained FDA approval in the US for reducing COPD exacerbations [122].

### Polyphenols

Polyphenols comprise a large group of natural antioxidant substances derived from plants. A common underlying feature of these molecules is the presence of one or more aromatic rings and at least one hydroxyl group. Several epidemiological studies have established a beneficial link between polyphenol intake and a lower risk of ROS-induced diseases. Resveratrol, a component of red wine, inhibits inflammatory cytokines in macrophages isolated from COPD patients. These anti-inflammatory effects may result from estrogen-like activity because of the structural similarity between resveratrol and steroids. Resveratrol also interacts with the thioredoxin pathway, thus preventing the redox-mediated activation of NF-κβ and AP1 [123]. Another well-studied polyphenol is curcumin, the active component of Curcuma longa, which is commonly known as turmeric. Like resveratrol, curcumin has been reported to inhibit NF-κβ activation, as well as IL-8 release, COX-2 expression, and neutrophil recruitment in the lungs. Recently, curcumin was shown to inhibit inflammation and restore glucocorticoid efficacy in response to oxidative stress. The anti-inflammatory actions of curcumin are likely propagated through the inhibition of histone acetyltransferase (HAT) activity and the prevention of NF-κβ-mediated chromatin acetylation [124]. The molecular mechanisms of the anti-inflammatory properties of dietary polyphenols targeting oxidative stress have not yet been well determined.

Taken together, various antioxidants which have been used or have potential to be used in clinics include the following categories: dietary vitamins, thiols, SOD mimetic, PDE4 inhibitors and polyphenols. Although dietary vitamins and polyphenols are very safe when used in human beings, there is still controversial about their significant benefit in chronic lung diseases. Among which, thiols and PDE4 inhibitors have promising effects both in improving oxidant-mediated cellular alterations and in beneficial observed in clinical trials. Increasing lung cellular levels of these antioxidants are critical in the enhancement of their bioavailability and potency in clinics. With a better understanding of the pathogenesis of oxidative stress in chronic lung diseases, other novel small molecular antioxidants with dual activities (antioxidant/anti-inflammatory) are expected to emerge soon.

## Conclusions

The evidence discussed in this review highlights the roles of ROS and the probable mechanism of ROS-induced mitochondrial dysfunction and mtDNA damage, as well as the crosstalk between AECs and AMs in the pathogenesis of lung degenerative, chronic inflammatory and tumorigenic diseases. Many targets identified in animal models still need to be investigated in humans [119, 125]. Further understanding of the effects of ROS-induced mitochondrial dysfunction on basic cellular functions, such as inflammation, immunological responses and repair mechanisms, will not only provide important information regarding these basic pathological processes but also identify more therapeutic targets for treating diseases [126, 127]. Many available antioxidants that could boost the endogenous antioxidant defense capabilities in humans are based on dietary vitamin and pharmacological agents. Recently, published data showed that antioxidant treatment may be a promising approach for improving ROS-induced epithelial dysfunction via promoting mitochondrial biogenesis in alveolar type II cells and transferring mitochondrial-containing bone marrow-derived stromal cells (BMSCs) [109, 128]. Developing new pharmacological, dietary and genetic approaches to increase lung antioxidant levels will be helpful for treating lung diseases that currently have no effective treatment options.

## Abbreviations

ROS: reactive oxygen species
mtDNA: mitochondrial DNA
O_2_^·−^: superoxide anion
^·^OH: hydroxyl radical
ONOO^−^: peroxynitrite molecule
SOD: superoxide dismutase
Cl^−^: chloride ion
Br^−^: bromide ion
HOCl: hypochlorous acid
HOBr: hypobromous acid
AMs: alveolar macrophages
ETC: electron transport chain
PASMCs: pulmonary artery smooth muscle cells
NAC: N-acetyl-l-cysteine
Tfam: mitochondrial transcription factor A
ΔΨm: mitochondrial membrane potential
DAMP: damage-associated molecular pattern
CCL2: CC chemokine ligand-2
AECs: alveolar epithelial cells
PINK1: PTEN-induced putative kinase 1
OGG1: 8-oxoguanine glycosylase
8-oxoG: 8-oxoguanine
mt-OGG1: mitochondria-targeted OGG1
NADPH oxidase: nicotinamide adenine dinucleotide phosphate oxidase
Nrf2: nuclear factor erythroid 2-related factor 2
ARE: antioxidant response element
PAI-1: plasminogen activator inhibitor-1
tPA: tissue plasminogen activator
uPA: urokinase
AT2: airway epithelial type 2
PKCδ: protein kinase delta
BAL: bronchoalveolar lavage
TBARs: thiobarbituric acid-reactive products
ALI: acute lung injury
ARDS: acute respiratory distress syndrome
ATP: adenosine triphosphate
GSH: glutathione
ELF: epithelial lining fluid
O_2_: oxygen
H_2_O_2_: hydrogen peroxide
COPD: chronic obstructive pulmonary disease
PDE4: phosphodiesterase type 4
cAMP: cyclic adenosine monophosphate
BMSCs: bone marrow-derived stromal cells
TCA cycle: tricarboxylic acid cycle
